# Comparative Proteomic Investigation of Plasma Reveals Novel Potential Biomarker Groups for Acute Aortic Dissection

**DOI:** 10.1155/2020/4785068

**Published:** 2020-03-18

**Authors:** Na Cheng, Hao Wang, Weizong Zhang, Heng Wang, Xiang Jin, Xiang Ma, Yitong Ma

**Affiliations:** ^1^The Department of Cardiology, First Affiliated Hospital of Xinjiang Medical University, Urumqi, 830054 Xinjiang, China; ^2^The Department of Health Care for Cadres, People's Hospital of Xinjiang Uygur Autonomous Region, Urumqi, 830001 Xinjiang, China; ^3^Shenzhen Omics Medical Research Center, Shenzhen, 518053 Guangdong, China; ^4^State Key Laboratory of Pathogenesis, Prevention and Treatment of High Incidence Diseases in Central Asia, Heart Center, First Affiliated Hospital of Xinjiang Medical University, Urumqi, 830054 Xinjiang, China

## Abstract

Acute aortic dissection (AAD) is a catastrophic cardiovascular disease with high disability and mortality due to multiple fatal complications. However, the molecular changes of the serum proteome after AAD are not very clear. Here, we performed isobaric tags for relative and absolute quantitation- (iTRAQ-) based comparative proteomic analysis to investigate the proteome profile changes after AAD by collecting plasma samples from 20 AAD patients and 20 controls. Out of the 345 identified proteins, 266 were considered as high-quality quantified proteins (95%confident peptides ≥ 2), of which 25 proteins were accumulated and 12 were reduced in AAD samples. Gene ontology enrichment analysis showed that the 25 AAD-accumulated proteins were enriched in high-density lipoprotein particles for the cellular component category and protein homodimerization acidity for the molecular function category. Protein-protein interaction network analysis showed that serum amyloid A proteins (SAAs), complement component proteins, and carboxypeptidase N catalytic chain proteins (CPNs) possessed the key nodes of the network. The expression levels of six selected AAD-accumulated proteins, B2-GP1, CPN1, F9, LBP, SAA1, and SAA2, were validated by ELISA. Moreover, ROC analysis showed that the AUCs of B2-GP1 and CPN1 were 0.808 and 0.702, respectively. Our data provide insights into molecular change profiles in proteome levels after AAD and indicate that B2-GP1 and CPN1 are potential biomarkers for AAD.

## 1. Introduction

Acute aortic dissection (AAD) is a catastrophic cardiovascular disease caused by injury of the innermost layer of the aorta, leading to vascular wall stratification by blood flows between the layers of the aortic wall [1, 2]. Although treatment guidelines have been well established, AAD has high fatality caused by irreversible vascular damage before the patient arrives at the hospital [3]. The risk of death increases by 1% to 3% per hour before surgery or medicinal treatment and can be as high as 21% for 24 hours and 74% for one week [4]. The key to reduce mortality is to shorten the time from symptom occurrence to receive appropriate treatment [5]. Combined with standard imaging methods using computed tomography angiography (CTA) and magnetic resonance angiography (MRA), quick diagnosis of AAD before the patients' arrival to the hospital using biomarkers with high sensitivity and specificity will greatly improve the survival rate and treatment guidelines [6–8]. However, most basic level hospitals do not have the equipment to perform CTA and MRA, which makes it more urgent to develop reliable early-stage biomarkers for AAD. Numerous potential biomarkers for diagnosis and prognosis of AAD were reported previously, such as D-dimer, microRNA-23a, magnitude soluble ST2, C-reactive protein (CRP), and smooth muscle myosin heavy chain (SM-MHC) [9–13]. However, all these potential biomarkers have disadvantages in specificity or sensitivity.

Proteomic technologies are widely used to profile the whole proteome expressed in specific specimens obtained from patients to determine potential biomarkers in various conditions [14–18]. Mass spectra- (MS-) based technologies have been greatly developed and are the most widely used proteomic approaches for their high throughput and sensitivity [19–21]. However, classic two-dimensional gel electrophoresis-based proteomic technologies are still irreplaceable since they provide a visualization map of the proteome and the truncation or modification information of protein isoforms [22–24]. Isobaric tags for relative and absolute quantitation (iTRAQ) technology is a high-efficiency nongel quantitative proteomic assay particularly suitable to identify biomarkers in low-abundance proteins [25–28]. Previous studies on acute aortic dissection reported several potential biomarkers including Lumican, C-reactive protein, and alpha 1 antitrypsin using iTRAQ and differential in-gel electrophoresis technologies [29, 30]. However, the repeatability, specificity, and sensitivity of these potential biomarkers in enlarged populations remain unclear.

Here, we performed an iTRAQ-based comparable proteomic analysis in the plasma samples obtained from AAD patients (*n* = 20) and controls (*n* = 20). Bioinformatic analysis of differenctially expressed proteins (DEPs) showed some enriched pathways related to AAD. Further validations of six selected AAD-accumulated proteins, B2-GP1, CPN1, F9, LBP, SAA1, and SAA2, were performed using an ELISA assay in a larger population (60 AAD samples and 50 control samples). Receiver operating characteristic curve (ROC) analysis showed that the area under curves of B2-GP1 and CPN1 were 0.808 and 0.702, respectively. Our study provided new evidence for potential biomarkers for acute aortic dissection.

## 2. Material and Methods

### 2.1. Clinical Samples

From January 2015 to February 2016, a total of 20 acute aortic dissection patients (AAD group) and 20 non-AAD patients (control group) who presented to the First Affiliated Hospital of Xinjiang Medical University (Urumqi, China) were enrolled. For the AAD group, whole blood samples were immediately collected in BD Vacutainer EDTA-2K tubes within 36 hours after onset. For the control group, blood samples were collected with limosis in the early morning, and the plasma was centrifuged at 5,000 rpm for 10 min at 4°C within 1 hour after collection. The plasma samples were then frozen and stored in aliquots at −80°C until protein extraction. Afterward, from March 2016 to February 2017, we continued to collect blood samples of 60 patients with acute aortic dissection within 72 hours after onset and 50 controls with normal coronary angiography as an expanded sample for ELISA. Inclusion criteria were as follows: (1) patients with acute aortic dissection should be confirmed by the aortic CTA or angiography; (2) non-AAD patients should be confirmed by coronary artery angiography. Exclusion criteria were as follows: (1) Marfan's syndrome, (2) recurrent aortic dissection, (3) familial aortic dissection, and (4) bicuspid aortic valve.

This study was carried out under the approval of the Medical Ethics Committee in the First Affiliated Hospital of Xinjiang Medical University (Approval No. 20160218-20). Written informed consents were obtained from all the participants prior to enrollment.

### 2.2. Protein Extraction and iTRAQ Analysis

The high-abundance proteins including albumin and IgG in plasma were depleted using a ProteoPrep® Immunoaffinity Albumin & IgG Depletion Kit (Sigma-Aldrich, Shanghai, China). Collected solutions were lysed in lysis buffer (7 M urea, 2 M thiourea, 50 mM Tris, 50 mM DTT, and 1 mM PMSF), and protein contents were determined using the Bradford assay (Bio-Rad, Beijing, China). Labeling for iTRAQ was performed according to the manufacturer's instruction (Applied Biosystems, Shanghai, China) and as previously described [31, 32]. The isotope tags 114, 115, and 116 were used to label the control group while 117, 118, and 119 were used for the AAD group.

### 2.3. Bioinformatic Analyses

All the identified proteins were mapped to gene ontology (GO) using BLAST2GO. The GO term enrichment analysis was performed by GOEAST [33]. The protein-protein interaction network was analyzed using STRING [34].

### 2.4. Verification of Representative DEPs

Six representative DEPs, including beta-2-glycoprotein 1 (B2-GP1), carboxypeptidase N catalytic chain (CPN), coagulation factor IX (F9), lipopolysaccharide-binding protein (LBP), serum amyloid A-1 protein (SAA1), and serum amyloid A-2 protein (SAA2), were selected and validated using an enzyme-linked immunosorbent assay (ELISA) kit (Wuhan ColorfulGene Biological Technology, Wuhan, China) in additional AAD (*n* = 60) and control (*n* = 50) samples.

### 2.5. Statistical Analysis

The iTRAQ data were analyzed using ProteinPilot 5.0 (AB Sciex, Shanghai, China) with the threshold of 95%confidential peptides ≥ 2 (unused score > 1.3). All statistical analyses were performed by SPSS 20.0. Different abundant proteins were determined when the *P* value was <0.05 and fold change was >1.5.

## 3. Results

### 3.1. Clinical Characteristics of Patients and Differential Abundant Proteins Identified by iTRAQ

The clinical characteristics of patients with acute artery dissection and the control group are shown in Table 1. No significant difference appears based on age or gender distribution between the AAD and control groups. However, as known risk factors to AAD, hypertension and smoking showed significant differences between the AAD and control groups (*P* < 0.05, Table 1, iTRAQ section).

**Figure 1:**
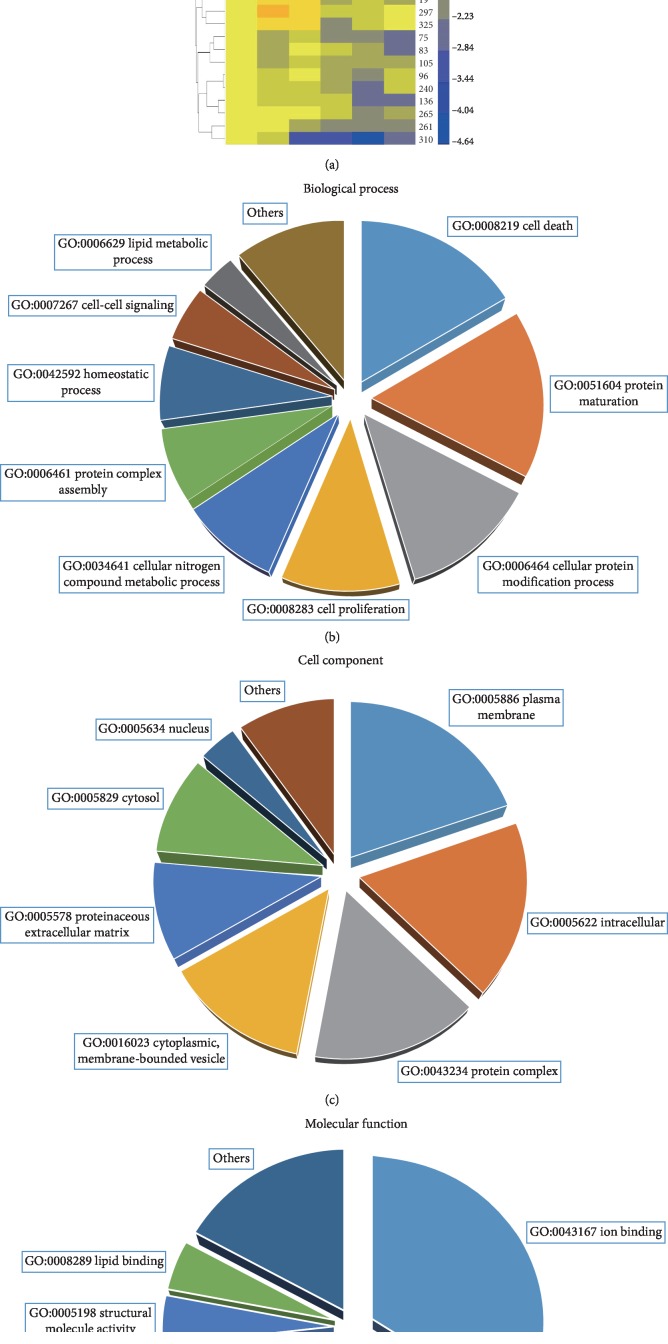
Heatmap and gene ontology classification of 37 differentially abundant proteins between AAD and normal controls. (a) The expression pattern of each protein in 3 pooled AAD patient samples (AAD 1–3) and 3 pooled normal control samples (control 1–3). The ID of the protein groups is listed on the right and the hierarchy cluster tree is shown on the left. Red boxes indicate higher expression level, blue indicates lower expression level, and yellow indicates the same expression level compared with the control. The gene ontology categories of biological process (b), cellular component (c), and molecular function (d). The GO ID and term for each section is indicated.

For the high-throughput mass spectra analysis, a total of 621,043 spectra were obtained, in which 208,353 were mapped to peptides in the database searched. In total, 266 high-quality quantified protein groups were identified out of 345 protein groups observed (95%confident peptides ≥ 2, see [Supplementary-material supplementary-material-1]). Furthermore, 25 and 12 proteins were determined as accumulated and reduced proteins, respectively (fold change > 1.5, 95%confident peptides ≥ 2), of which detailed information is listed in Table 2. The expression patterns of 37 different abundant proteins are shown in Figure 1(a). The GO term distribution of biological processes, cellular components, and molecular functions was also analyzed. The GO:008219 (cell death), GO:0051604 (protein maturation), and GO:0006464 (cellular protein modification process) are the most abundant GO terms of the biological process category (Figure 1(b)). The GO:0005886 (plasma membrane), GO:0005622 (intracellular), and GO:0043234 (protein complex) are the most abundant GO terms of the cellular component category (Figure 1(c)). The GO:0043167 (ion binding), GO:0030234 (enzyme regulator activity), and GO:0008233 (peptidase activity) are the top three GO terms of the molecular function category (Figure 1(d)).

### 3.2. Gene Ontology Enrichment and Protein-Protein Interaction Networks

**Figure 2:**
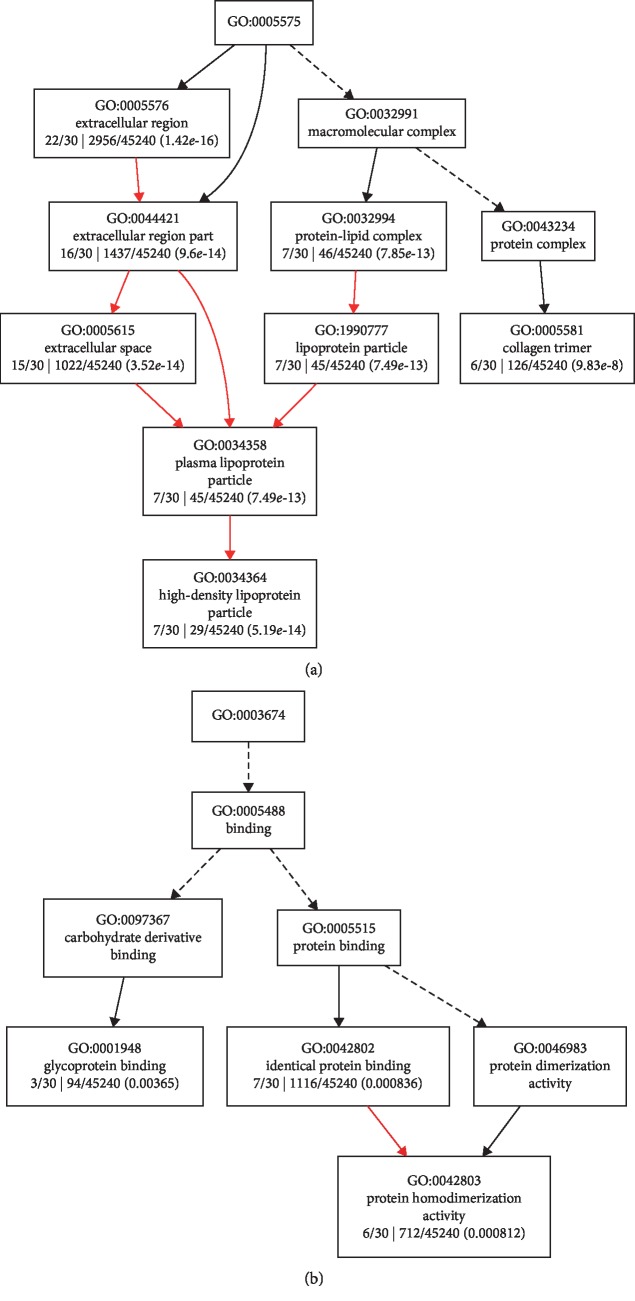
Enrichment analysis of 25 induced proteins. Tree view of gene ontology cellular component (a) and molecular function (b) categories. GO terms are represented by boxes. Yellow boxes indicate significant GO terms. The FDR *P* values and numbers of DEPs for each GO term are also shown, and arrows are used to connect GO terms. Black dashed arrows indicate two nonsignificant GO terms; red arrows indicate two significant GO terms; black solid arrows indicate one significant and one nonsignificant GO term.

To better understand the biological pathways that the DEPs involved, GO enrichment analysis was performed using Gene Ontology Enrichment Analysis Software Toolkit (GOEAST) [33]. Tree views of the cellular component (Figure 2(a)) and molecular function (Figure 2(b)) categories in which the 25 AAD-induced proteins were enriched are shown. The GO terms of high-density lipoprotein particle (GO:0034364) and collagen trimer (GO:0005581) were the branch-ends for the cellular component category, and glycoprotein binding (GO:0001948) and protein homodimerization activity (GO:0042803) were the branch-ends for the molecular function category. The tree view of the biological process category in which the 25 AAD-induced proteins were enriched is also provided as Supplementary [Supplementary-material supplementary-material-1].

**Figure 3:**
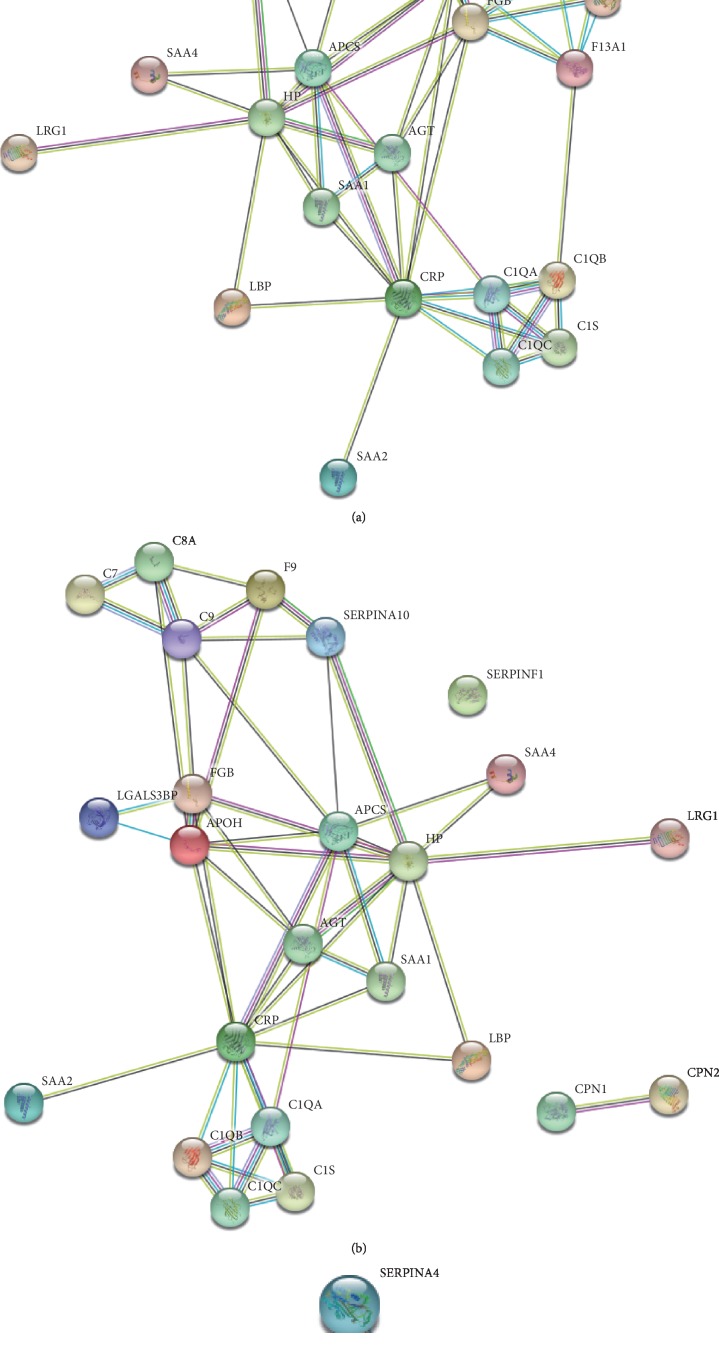
Protein-protein interaction network analysis of 37 differentially abundant proteins (a), 25 AAD-induced proteins (b), and 12 AAD-reduced proteins (c). Colored lines between the proteins indicate the various types of interaction evidence. Protein nodes which are enlarged indicate the availability of 3D protein structure information.

Moreover, protein-protein interaction (PPI) networks of the total 37 DEPs (Figure 3(a)), as well as the 25 AAD-induced proteins (Figure 3(b)) and 12 AAD-reduced proteins (Figure 3(c)), were shown. The PPI of AAD-induced proteins showed that serum amyloid A proteins (SAAs), complement component proteins, and carboxypeptidase N catalytic chain proteins (CPNs) possessed the key nodes of the network (Figure 3(b)), indicating that these proteins may be important for AAD development.

### 3.3. Potential Biomarker Groups Validated by ELISA in Extended Samples

According to the bioinformatic analyses of the DEPs, several representative proteins were selected as potential biomarkers for AAD to perform further validation using an ELISA assay in extended samples. The selected potential biomarker candidates include beta-2-glycoprotein 1 (B2-GP1), carboxypeptidase N catalytic chain (CPN), coagulation factor IX (F9), lipopolysaccharide-binding protein (LBP), serum amyloid A-1 protein (SAA1), and serum amyloid A-2 protein (SAA2), which are included in significantly enriched GO terms and protein-protein interaction network nodes (Figures 2 and 3). The clinical characteristics information of the 110 (60 AAD and 50 controls) extended patients and control samples is provided in the ELISA section in Table 1.

**Figure 4:**
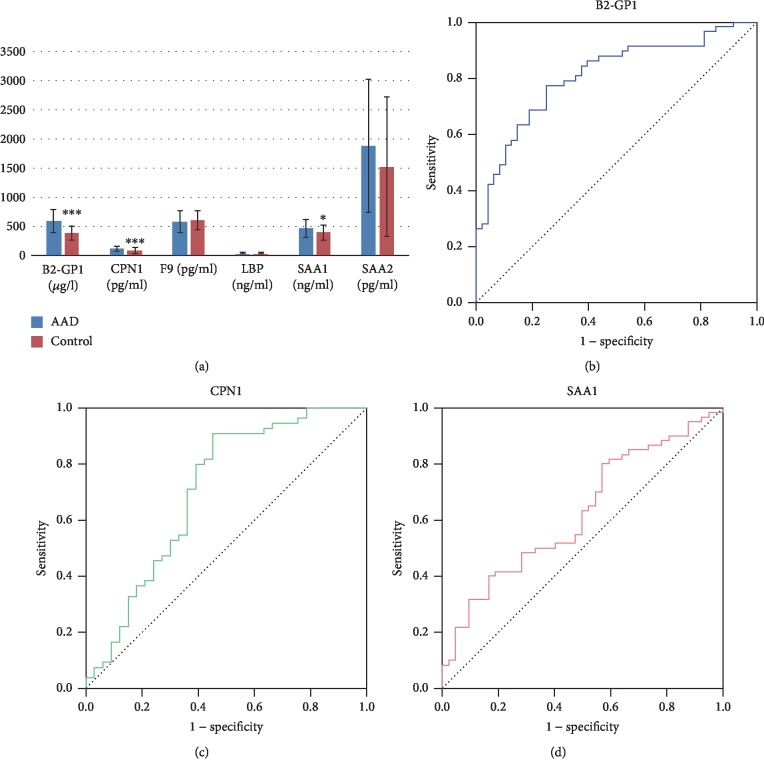
ELISA and receiver operating characteristic (ROC) curve analyses using the serum levels of B2-GP1, CPN1, F9, LBP, SAA1, and SAA2 in AAD patients and normal controls. (a) ELISA assays were performed to validate the serum levels of six DEPs in expanded samples of AAD patients and controls. ROC curves of B2-GP1, CPN1, and SAA1 are shown in panels (b), (c), and (d), respectively. The AUCs of B2-GP1, CPN1, and SAA1 are 0.808, 0.702, and 0.623, respectively.

The contents of the candidate proteins in the plasma samples are calculated and shown in Figure 4(a). Significantly different amounts of B2-GP1 (*P* < 0.001), CPN1 (*P* < 0.001), and SAA1 (*P* < 0.05) were found between the control (blue columns) and AAD (orange columns). Furthermore, the receiver operating characteristic (ROC) curves of the 3 biomarker candidates with significant differential amounts between AAD patients and controls are illustrated (Figures 4(b)–4(d)) to assess their abilities to diagnose AAD. As is shown, two candidates including beta-2-glycoprotein 1 (B2-GP1) and carboxypeptidase N catalytic chain (CPN1) exhibited significant specificities to AAD, with the AUC values of 0.808 and 0.702, respectively. The ELISA and ROC curve data indicated that B2-GP1 and CPN1 could potentially serve as novel biomarkers to facilitate the diagnosis of AAD at an early stage. A further investigation of B2-GP1 and SAA1 in patients that recovered from surgery was also performed, resulting in nonsignificant levels of these two proteins between controls and cured patients (Supplementary [Supplementary-material supplementary-material-1], *n* = 23).

## 4. Discussion

The morbidity of AAD increased during recent years accompanied with a high frequency of hypertension and other chronic diseases [35]. About 3/4 of patients are older than 40 years old, and the incidence of male patients is 2–3 times higher than that of women of the same age. Even with modern imaging methods (such as computed tomography and magnetic resonance), AAD is still often overlooked or misdiagnosed often owing to variability in presentation and lack of suspicion by the attending physician, leading to high mortality due to potentially fatal complications with a mortality rate of approximately 1% per hour within the first 24 h after symptom onset [36]. The 24-hour mortality risk is about 21%, and the risk of death within 1 week is about 74%, while the 1-year mortality rate is 93% [37, 38]. To develop novel plasma biomarkers that are more effective and specific to AAD will be helpful for reducing the mortality of AAD, developing individualized treatment for AAD patients, and improving prognosis.

As a high-throughput proteomic technology developed in recent years, iTRAQ technology is an effective method to develop novel serum markers and has been used to identify plasma protein markers for various diseases [39–43]. Previous studies reported potential AAD biomarker candidates discovered by iTRAQ technology [29, 30]. However, limited by the resolution of mass spectrometry, Gu et al. identified only 174 proteins from pooled serum samples, but the different abundant proteins are not reliable due to lack of biological and technical replicates [29]. Likely, Xiao et al. identified 355 proteins, while only 164 proteins reached the strict quantitative standard. The threshold to determine significantly different abundant proteins was not strict (1.2-fold), leading to 125 different abundant proteins out of 164 high-quality quantitative proteins (over 76% proteins are differentially accumulated) [30]. The potential biomarkers reported by these previous works are Lumican, D-dimer, CRP, and TSP-1. However, a single biomarker can only respond to a moment of some physiological and pathological state and is unable to reflect the physiological state of multiple pathologies. These biological markers have poor specificity, as the expression levels were different in various chest diseases. For instance, D-dimer is reported to increase not only in AAD but also in acute myocardial infarction, angina pectoris, and pulmonary embolism disease [44, 45]. We carried out an experiment including pooled biological replicates, as well as technical replicates by labeling samples of each group with three different isobaric tags (114, 115, and 116 for the control group; 117, 118, and 119 for the AAD group). In addition, an advanced AB Sciex TripleTOF 5600 plus system was used to perform the iTRAQ experiment, resulting in 345 identified proteins and 266 high-quality quantified proteins (the most high-quality data of plasma proteomics in AAD to date). A total of 37 different abundant proteins (25 increased and 12 reduced) were identified due to biological and technical replicates, leading to a high reliability of protein expression levels (Figure 1 and Table 2).

The pathway enrichment analysis of the AAD-induced proteins showed that high-density lipoprotein particles and collagen trimers were the branch-ends of significantly enriched GO terms for the cellular component category (Figure 2(a)). This is consistent with the knowledge that high plasma lipid/cholesterol is a risk factor for cardiovascular diseases. Furthermore, the PPI analysis showed that serum amyloid A proteins (SAAs), complement component proteins, and carboxypeptidase N catalytic chain proteins (CPNs) possessed the key nodes of the network (Figure 3(b)), indicating the potential possibility of these proteins to serve as biomarkers. Thus, six of these proteins were selected to be examined in an expanded group of AAD and control plasma samples. Previous proteomic investigation in AAD identified ten significantly differentially expressed proteins between AAD and controls [30], including complement component C9, Lumican, Ceruloplasmin, complement factor B, and CRP, which also showed differential expression levels in this study (Table 2). The ELISA results showed that B2-GP1 and CPN1 have significant ROC curves, indicating the potential possibility of these two proteins as biomarkers (Figures 4(b) and 4(c)). The B2-GP1 is a phospholipid-binding glycoprotein and was reported to be involved in the immune system by directly interacting with membrane Toll-like receptors, resulting in activation of endothelial cells and monocytes and expression of proinflammatory cytokines [46]. The B2-GP1 also has been reported as a potential biomarker for predicting thrombosis after renal transplantation [47]. CPN1 was also reported to be useful in early detection of breast cancer [48]. These independent works suggest that B2-GP1 and CPN1 are involved in human disorders and may serve as potential biomarkers.

In summary, our work provides a comprehensive proteomic investigation for molecular changes after AAD. For the 25 AAD-induced proteins, significantly enriched GO terms of high-density lipoprotein particles and collagen trimers were identified. From the PPI key nodes, six AAD-induced proteins were selected for validation in an expanded group of samples. The ELISA and ROC curve analysis showed that B2-GP1 and CPN1 are significantly accumulated in the plasma of AAD patients and may be potential biomarkers for detecting AAD early.

## Figures and Tables

**Table 1:** Clinical features of subjects used for iTRAQ and ELISA analysis.

iTRAQ	AAD	Control	*P* value
*n*	20	20	—
Age (mean (± SD))	47.05 ± 8.01	52.15 ± 11.14	0.105^a^
Female (*n* (%))	4 (20.0)	6 (30.0)	0.465^b^
Male (*n* (%))	16 (80.0)	14 (70.0)	0.465^b^
Stanford type A	0	—	—
Stanford type B	18	—	—
Intramural hematoma	2	—	—
Hypertension (*n* (%))	15 (75.0)	8 (40.0)	0.025^b^
Diabetes mellitus (*n* (%))	1	0	—
Smoking (*n* (%))	14 (70.0)	7 (35.0)	0.027^b^
TC (mean (± SD))	4.19 ± 1.11	3.68 ± 0.93	0.130^a^
LDL-C (mean (± SD))	2.43 ± 0.76	2.26 ± 0.83	0.533^a^
HDL-C (mean (± SD))	1.15 ± 0.36	1.09 ± 0.19	0.556^a^
TG (mean (± SD))	2.04 ± 1.91	1.59 ± 1.04	0.375^a^

ELISA	AAD	Control	*P* value
*n*	60	50	—
Age (mean (± SD))	51.43 ± 10.52	53.44 ± 10.33	0.317^a^
Female (*n* (%))	10 (16.7)	8 (16.0)	0.925^b^
Male (*n* (%))	50 (83.3)	42 (84.0)	0.925^b^
Stanford type A	5	—	—
Stanford type B	45	—	—
Intramural hematoma	10	—	—
Hypertension (*n* (%))	46 (76.7)	24 (48.0)	0.002^b^
Diabetes mellitus (*n* (%))	3 (5.0)	0	—
Smoking (*n* (%))	32 (53.3)	26 (52.0)	0.889^b^
TC (mean (± SD))	4.27 ± 0.93	3.82 ± 0.84	0.010^a^
LDL-C (mean (± SD))	2.67 ± 0.70	2.42 ± 0.78	0.079^a^
HDL-C (mean (± SD))	1.15 ± 0.30	1.03 ± 0.19	0.010^a^
TG (25%–75% percentiles)	0.82~1.89	0.89~1.93	0.588^c^

^a^ *t*-test. ^b^Chi-square test. ^c^Mann-Whitney *U* test. TC: total cholesterol; LDL-C: low-density lipoprotein-cholesterol; HDL-C: high-density lipoprotein cholesterol; TG: triglyceride.

**Table 2:** Detailed information of 25 accumulated and 12 reduced proteins in artery dissection patients.

*N*	Name	95% pep.^∗^	Fold	*P* value
11	Haptoglobin	396	3.29	0.0113
35	Complement component C9	41	2.51	0.0161
39	Complement component C7	38	1.72	0.0395
43	Serum amyloid A-2 protein	86	8.07	0.0003
50	Beta-2-glycoprotein 1	61	1.73	0.0429
53	Complement C1s subcomponent	37	1.94	0.0186
57	Pigment epithelium-derived factor	27	1.98	0.0091
58	Complement factor I	28	2.02	0.0274
63	Complement component C8 alpha chain	25	1.55	0.0337
72	Galectin-3-binding protein	14	1.92	0.0055
76	Leucine-rich alpha-2-glycoprotein	15	4.06	0.0035
77	Angiotensinogen	28	3.01	0.0052
79	Carboxypeptidase N subunit 2	18	1.83	0.0371
81	Serum amyloid P component	15	2.24	0.0065
84	Carboxypeptidase N catalytic chain	10	2.13	0.0174
101	Coagulation factor IX	13	1.82	0.0003
116	C-reactive protein	12	6.57	0.0002
117	Complement C1q subcomponent subunit B	18	2.14	0.0289
118	Protein Z-dependent protease inhibitor	8	1.73	0.0048
120	Complement C1q subcomponent subunit A	11	1.98	0.0070
128	Lipopolysaccharide-binding protein	7	2.99	0.0055
133	Serum amyloid A-4 protein	14	1.77	0.0127
151	Serum amyloid A-1 protein	113	5.86	0.0015
152	Complement C1q subcomponent subunit C	10	2.23	0.0028
252	Fibrinogen beta chain	3	1.87	0.0065

*N*	Name	95% pep.	Fold	*P* value
19	Isoform 10 of fibronectin	63	0.28	0.0004
75	Histidine-rich glycoprotein	22	0.32	0.0111
83	Kallistatin	11	0.51	0.0381
96	Ig kappa chain V-II region RPMI 6410	76	0.58	0.0414
105	Peroxiredoxin-2	7	0.54	0.0492
136	Coagulation factor XIII A chain	6	0.45	0.0309
240	eIF 4 gamma 3	26	0.52	0.0239
261	Reversed carbonic anhydrase 1	2	0.27	0.0109
265	Ig kappa chain V-III region POM	86	0.58	0.0231
297	Cartilage oligomeric matrix protein	2	0.61	0.0308
310	Isoform 2 of chondroadherin-like protein	2	0.19	0.0420
325	A-kinase anchor protein 7 isoform gamma	2	0.55	0.0292

^∗^Numbers of 95% confidential peptides.

## Data Availability

The mass spectrometry proteomic data have been deposited to the ProteomeXchange Consortium (http://proteomecentral.proteomexchange.org) via the iProX partner repository [49] with the dataset identifier PXD013465.

## Supplementary Materials

Supplementary 1 Table S1: detailed information of all identified serum proteins from artery dissection patients and normal controls.

Supplementary 2 Figure S1: tree view of enrichment analysis for 25 induced proteins in the biological process category.

Supplementary 3 Figure S2: ELISA analyses of B2-GP1 and SAA1 in normal controls and cured AAD patients.
