# Supplementary figures and images for: Comparative Proteomic Investigation of Plasma Reveals Novel Potential Biomarker Groups for Acute Aortic Dissection
Source: Dis Markers. 2020 Mar 18;2020:4785068. doi: 10.1155/2020/4785068 (PMC7106916; doi:10.1155/2020/4785068)

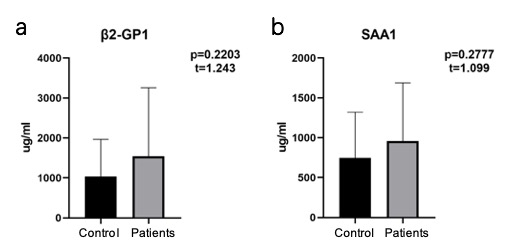

Supplement: Supplementary 3 — Figure S2: ELISA analyses of B2-GP1 and SAA1 in normal controls and cured AAD patients. [file 4785068.f3.jpg]
